# Luminescent Behavior of Gels and Sols Comprised of Molecular Gelators

**DOI:** 10.3390/gels7010019

**Published:** 2021-02-17

**Authors:** Girishma Grover, Richard G. Weiss

**Affiliations:** Department of Chemistry, Institute for Soft Matter Synthesis and Metrology, Georgetown University, Washington, DC 20057, USA; gg603@georgetown.edu

**Keywords:** gel, luminescence, molecular organic gelator (LMOG), hydrogel, fluorescence, phosphorescence, aggregation-induced emission enhancement (AIEE), quantum yield, excimer

## Abstract

We present a brief review of some important conceptual and practical aspects for the design and properties of molecular luminescent gelators and their gels. Topics considered include structural and dynamic aspects of the gels, including factors important to their ability to emit radiation from electronically excited states.

## 1. Introduction

Molecular organic gelators (LMOGs) and gels made from them are an important class of molecules/materials with potential and realized applications in the fields of drug delivery and tissue engineering [1], oil spill recovery [2], self-healing [3], and 3D printing [4]. Although multiple examples of luminescent gels are discussed in this review, our focus lies particularly on the constituent LMOGs. In that regard, we will not cover fundamental aspects of photochemistry and photophysics or basic experimental details related to them because they can be found in many excellent texts [5,6,7,8,9,10,11]. Additionally, we do not provide a comprehensive overview of all of the examples of luminescent gels found in the literature or circularly polarized emission from LMOG assemblies [12,13,14]. In fact, most are fluorescent; very few have been reported to be phosphorescent. Rather, we discuss the types of luminescent gels based on the inherent luminescence exhibited by gelator molecules depending upon the type and position of the emitting functional group present, the effects of the position and length of alkyl spacers and the effects of adding an external lumophore to the gel matrix. Even then, it is difficult to derive *global conclusions* about the relationships between LMOG structure and efficiency of gel emission. Extremely subtle changes in the former have been shown in several examples to cause enormous changes in the latter. Furthermore, it is difficult to predict whether the homologue of a known LMOG will even form a gel or whether an LMOG known to form a gel in one solvent will form one in a seemingly similar solvent [15,16].

Another criterion for the classification of luminescent gels is the ability of the LMOGs either to enhance or to reduce the emission intensity when a sample exists in its solution, sol, or gel state. We also discuss some of the important properties of luminescent gels, such as their critical gelator concentrations (CGCs), reproducibility, and thermal and temporal stabilities, as well as how their steady state and dynamic emission characteristics are affected by molecular packing within the fibrils or other multi-molecular units that constitute the gel assemblies.

LMOGs are capable of forming one-dimensional anisotropic fibrils which can aggregate further into three-dimensional self-assembled networks through covalent or non-covalent interactions (such as hydrogen bonding, van der Waals and London dispersion forces, hydrophobic interactions, π–π stacking or coordination bonding) [15,16]. The major component of gels by weight and mole fraction is solvent molecules. They are immobilized macroscopically (but not microscopically) with respect to flow within the gels by capillary forces, surface tension, and related surface–solvent interactions. In that respect, the resultant materials are solid-like and termed gels depending on their rheological properties [17]. Additional information about the dependence of gelation on solvent has been analyzed in the literature [18,19]. The degree of solubility of gelators in a solvent is a crucial factor in determining whether a gel will be formed (i.e., a micro-phase separated 3D-network of the gelator within a liquid). Too great solubility will deter intermolecular interactions among gelator molecules and, thus, inhibit self-assembly. At the other extreme, the inadequate solubility of a gelator does not permit the in situ intermolecular interactions necessary for a gel network to be formed [20]. Thus, balanced solvent–gelator interactions are essential for gel formation. Depending on its structure, a gelator may gelate low or high polarity organic liquids (or both), thus forming “organogels”. Gels formed in water or aqueous media are commonly referred to as “hydrogels”. Hydrogels, especially, have found many applications in biological systems [21]. In some cases, the addition of a small amount of water to an organic liquid can increase the probability of forming a gel network due to the initiation of strong intermolecular H-bonding [22]. 

Many gels respond to external stimuli, such as heat, mechanical perturbations, sonication, pH, and light. In this way, they can be thermo-reversible, thixotropic, acid-base sensors, etc., in ways that affect their luminescent properties. As a result, these gels have been studied widely for applications in diverse fields, such as excitation energy transfer and light harvesting [23,24,25,26], dye-sensitized solar cells [27], sensing of metal ions and explosives [28,29,30,31], acid and base sensing [32,33], and as templates for nanoparticle synthesis [34,35,36].

**Figure 1:**
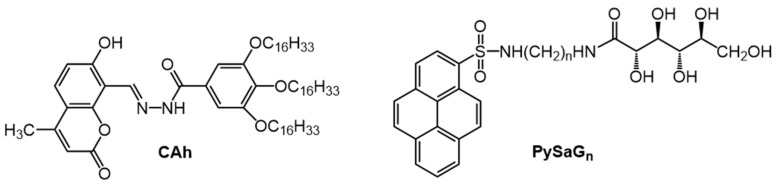
Molecular structures of CAh and PySaG_n_ (where n = 2,3,4,6,7,8) gelators.

For example, Hu et al., designed an ionic, stimulus-responsive fluorescent gel using the long alkyl-chain coumarin acylhydrazone, CAh, as the LMOG (Figure 1) [37]. Because of their luminescent properties, some coumarin derivatives are known to be able to act as sensors of ions [38]. Thus, 3% CAh in n-BuOH:water (*v*:*v*, 9:1) forms a fluorescent gel that can selectively detect as low as 3.88 × 10^−8^ M of sulphide ions (S^2−^) and 1.64 × 10^−8^ M of cyanide ions (CN^−^) ions in water by exhibiting a blue shift in its emission when excited at 340 nm.

**Figure 2:**
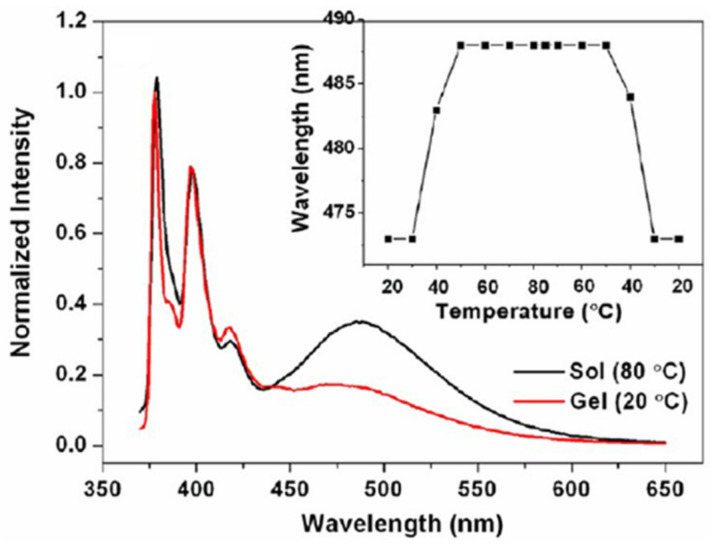
Fluorescence spectra (λ_ex_ = 350 nm; intensity normalized at 378 nm) of 1.5% (2.6 × 10^−2^ M) PySaG_7_ in acetonitrile in the gel (20 °C) and sol (80 °C; gel melting temperature, T_gel_ = 79 °C) phases. The inset shows the temperature-dependent wavelength maxima of the excimeric emission. Reprinted (adapted) with permission from [39]. Copyright (2013) American Chemical Society.

Fluorescent LMOGs can also be used to probe the gelation process, degree of aggregation, and nature of the molecular assembly process within the gel network. In one such study, Yan et al., used gelators comprised of a 1-pyrenyl group with a glucono substituent and diamine spacers of different lengths, PySaG_n_ (Figure 1) [39]. In acetonitrile with excitation at 350 nm, a 10 nm blue shift and lowered intensity of excimeric emission were observed in the gel form (λ_em_ = 478 nm, 20 °C) of 1.5% PySaG_7_ compared to the sol (λ_em_ = 488 nm, 80 °C) (Figure 2). This change suggests the easier reorientation of pyrenyl groups of PySaG_7_ in the sol, facilitating excimer emission. In the gel phase, the more restrictive packing arrangement makes the attainment of the excimer geometry more difficult. 

### 1.1. Following the Energy Flow

There are multiple ways by which excited state energy can be used through radiative or non-radiative processes to return a molecule to a ground state species. The potential routes can be categorized by Jablonski diagrams [40]. Non-radiative processes include internal conversion, intersystem crossing and reactions to photoproducts. In this review, we focus on the processes that involve radiative transitions, which include fluorescence, phosphorescence, and delayed fluorescence (DF). Non-radiative processes are discussed only insofar as they influence the rates and efficiencies of the radiative processes. Specifically mentioned is information concerning the lifetimes (τ) and quantum efficiencies (φ) of the excited states. 

#### 1.1.1. Phosphorescence

**Figure 3:**
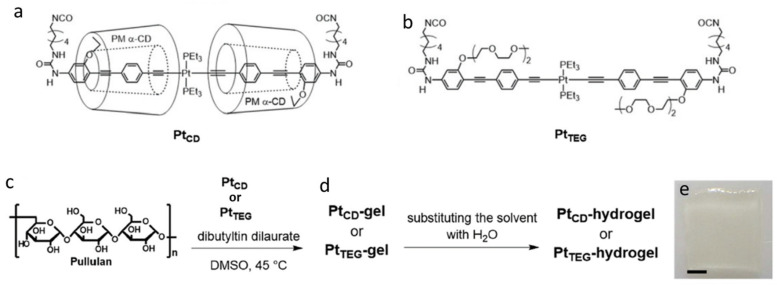
Molecular structure of (**a**) Pt_CD_; (**b**) Pt_TEG_; (**c**) and (**d**) formation of a Pt_CD_-hydrogel and Pt_TEG_-hydrogel and (**e**) photograph of the Pt_CD_-hydrogel in daylight; scale bar = 1 mm. Republished from [42] with permission of Royal Society of Chemistry.

The generally longer lifetimes of phosphorescence than fluorescence allow many triplet states to be quenched easily through collisions with solvent molecules or molecular oxygen dissolved within a liquid [41]. Usually, quenching of triplet states is addressed by following the emission of metal ions or by adding a phosphor that emits after energy transfer from a non-phosphorescent species in the gel. One example is the introduction of a spatially isolated Pt-acetylide crosslinker in an oligo(phenylene ethynylene), Pt_TEG_, to obtain a phosphorescent hydrogel [42]. In this case, permethylated α-cyclodextrins (PMα-CDs) were added to form a gelator "insulated" from external contact quenchers, Pt_CD_ (Figure 3). Aggregation of the π-conjugated system is hindered sterically by PMα-CD, increasing the overall phosphorescent quantum yield. In a nitrogen atmosphere and using excitation at 375 nm, the phosphorescence quantum yield, φ_P_, for the Pt_CD_-hydrogel was 9.9% (λ_max_ = 533 nm); in the presence of air or in the absence of PMα-CD (Pt_TEG_-hydrogel), the φ_P_ dropped to less than 1%. 

In another example, a phosphorescent gel was constructed by placing the organic phosphor, 3,5-dibromoquinoline (BrQ), into a N,N-dibenzoyl-L-cystine matrix (DBC) [43]. The gel system (BrQ-DBC) consisted of 5 × 10^−4^ M BrQ and 0.6 wt % DBC in DMSO:water (1:9). The gel both fluoresces at 367 nm (τ_F_ = 1.5 ns) and phosphoresces at 550 nm (τ_P_ = 280 μs) when excited at 315 nm. BrQ alone in DMSO displayed no phosphorescence. Thus, the entrapping of BrQ in the ordered BrQ-DBC-gel matrix suppressed the quenching of the triplet state. Additionally, the DBC-BrQ gel was found to be thermally responsive, and sensitive to both pH changes (in the presence of carboxylic acids), and to redox processes (upon addition of disulfide groups). Sols from these gels regained most of their dual emissive properties when reconverted to their gel phases. 

Although numerous examples of fluorescent LMOGs have been reported, few examples of phosphorescent LMOGs and gels are known. An interesting example of a phosphorescent LMOG producing gels was found by Zhang et al., using an organic molecule with an α-diketo group at the 9,10 positions along an 18-carbon fatty acid chain, DODA [44]. Details are discussed in Section 3 and Section 5.

#### 1.1.2. Delayed Fluorescence (DF)

In delayed fluorescence (DF), a molecule in its T_1_ excited state undergoes reverse intersystem crossing to become an S_1_ state from which it undergoes radiative decay. There are two types of delayed fluorescence: thermally activated (E-type) and triplet–triplet annihilation (P-type). 

**Figure 4:**
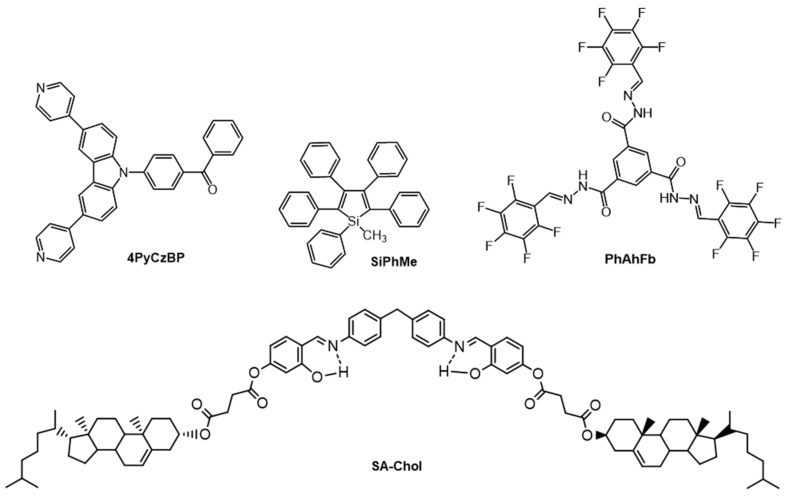
Molecular structures of 4PyCzBP, SiPhMe, PhAhFb and SA-Chol.

By definition, thermally activated delayed fluorescence (TADF) requires overcoming an energy barrier between an S_1_ state and a lower energy T_1_ state. The emission spectra for molecules undergoing this type are the same as that of prompt fluorescence. E-type delayed fluorescence was first found with eosin [45]. Both prompt and TADF were documented in dichloromethane solutions of a pyridinyl-carbazole-based molecules, 4PyCzBP (Figure 4) [46]. When one equivalent of L-tartaric acid was added, a gel formed due to H-bonding interactions between a carboxylic acid group and a nitrogen atom of a pyridinyl group; the CGC value was 3 mg/mL. The 4PyCzBP emits at 477 nm (φ_F_ = 52%) in solutions of deoxygenated dichloromethane, while the 4PyCzBP-tartaric acid gel emits at 510 nm (φ_F_ = 36%). This suggests a more defined packing arrangement in the gel phase. Because φ_F_ is only 10% for 4PyCzBP in aerated dichloromethane, the authors conclude that the quenching of emission occurred from a triplet state of 4PyCzBP. In these solutions, with λ_ex_ = 378 nm, τ_F_ was 33.5 ns and two TADF decay times, τ_DF_ = 0.61 and 6.32 μs, were detected; in the gel phase (i.e., in the presence of tartaric acid), τ_F_ = 20.0 ns and only one TADF component, τ_DF_ = 2.30 μs, was observed. This metal-free self-assembled network may offer an inexpensive alternative to phosphorescent organic light emitting diodes [47]. 

P-type annihilation involves the transformation of two triplet state molecules [48,49] to form an excimer or excited monomers. In this case, the wavelength of the delayed fluorescence is usually between those of the normal fluorescence and phosphorescence [50]. The intensity of this type of DF is also very concentration dependent. It commonly occurs in solutions of aromatic hydrocarbons [51].

## 2. Types of Fluorescent and Phosphorescent Gels 

### 2.1. Fluorescent Gels 

Fluorescent gels frequently can be placed in one of two categories: (1) the gelator fluoresces more strongly in a gelled state than in solutions/sols or (2) the gelator emits in solution/sol states but is quenched in the gelled state.

#### 2.1.1. Gelator Fluoresces More Strongly in a Gelled State Than in Solutions/Sols

Aggregation induced emission enhancement (AIEE) is the term used when the gelator does not emit (or is weakly emitting) in its solution or sol but emits strongly in the gel states [52,53]. Xue et al., reported a 1000-fold increase in fluorescence emission intensity when a sol of an LMOG containing salicylidene-aniline and cholesterol units (SA-Chol, Figure 4) in benzene was converted to its gel phase by adding cyclohexane [54]. This increase could be attributed to the formation of aggregates in which molecular motion within the gelator network was restricted (because the LMOG concentration was kept constant throughout).

**Figure 5:**
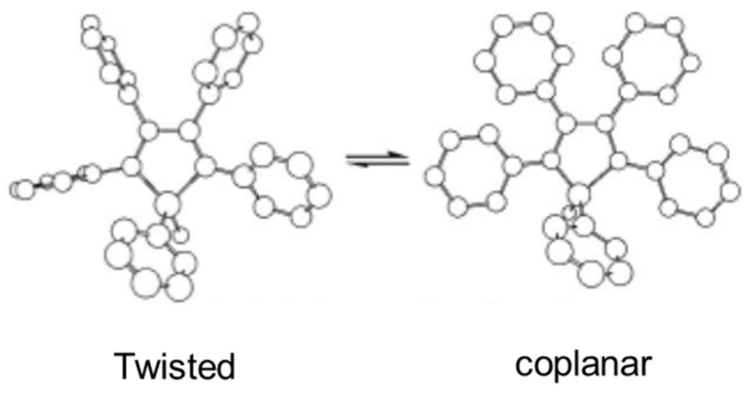
Conformational rotamers of SiPhMe. Republished from [55] with permission of Royal Society of Chemistry.

This AIEE phenomenon has been observed in 1-methyl-1,2,3,4,5-pentaphenylsilole, SiPhMe (Figure 4) [55]. In 10 µM ethanol solution, it emitted very weakly when excited at 381 nm. However, it aggregated in a 90:10 water:ethanol mixture and fluoresced strongly at ~500 nm. The very low φ_F_ in ethanol, 0.63 × 10^−3^, increased to 0.21 in the water:ethanol mixture. This increase has been attributed to restrained conformational motion by rotamers in the gel. As shown in Figure 5, SiPhMe can exist in a twisted conformer (twisted peripheral phenyl groups) in solution, while planarity (a coplanar conformer) is induced upon aggregation. Because the degree of conjugation is maximized in the planar structure, the aggregated state results in enhanced fluorescence intensity, as well as red shifts in the absorption and emission bands. On the other hand, the steric crowding and co-facial assembly of SiPhMe in the solution state inhibits excimer formation. 

The temporal dependence of gel formation can also be monitored using changes in the AIEE. Ma et al., studied AIEE of the gelator, PhAhFb, that contains both acylhydrazone and fluorobenzene groups (Figure 4) [56]. The aggregation of PhAhFb was favored by its H-bonding sites and enhanced further by coordination with added metal ions. As an added benefit, the coordination reduced radiationless decay of the excited singlet state by constraining intramolecular rotational and vibrational motions. Thus, AIEE was observed upon excitation at 380 nm when Al^3+^ ions were added to a gel of 1.3% PhAhFb in a 1:2 DMSO:ethylene glycol mixture. The 1:1 (PhAhFb:Al^3+^) complex exhibited a greater than 10-fold increase in fluorescence intensity and a blue shift of 32 nm (489 to 457 nm) compared to the native gel. 

**Figure 6:**
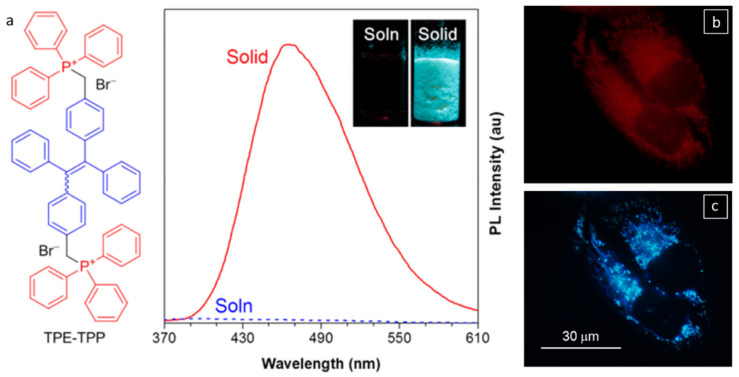
(**a**) Molecular structure of TPE-TPP; photo-luminescence spectra of TPE-TPP in solid and solution states. Inset: Photographs of a DMF solution (left) and a solid powder (right) of TPE-TPP taken under UV radiation. Concentration of TPE-TPP = 10 μM; λ_ex_ = 321 nm. Fluorescent images of CCCP (10 μM) treated HeLa cells stained with (**b**) MT (50 nM) for 15 min and (**c**) TPE-TPP (5 μM) for 30 min. λ_ex_ = 540−580 nm (for MT) and 330−385 nm (for TPE-TPP). Reprinted (adapted) with permission from [57]. Copyright (2013) American Chemical Society.

Leung et al., utilized AIEE from a tetraphenylethene (TPE) gelator with triphenylphosphonium groups (TPE-TPP, Figure 6a) to study mitochondria-mediated apoptosis [57]. The high photostability of TPE-TPP upon aggregation was attributed to photobleaching of the outer layer of aggregate molecules that prevented the destruction of molecules located inside the aggregates of TPE-TPP. As such, TPE-TPP has some advantages for AIEE over some more commonly used dyes, such as MT (MitoTracker Red FM) [58]. Thus, HeLa cells stained with 5 μM TPE-TPP for 1 h retained 80% of their fluorescence emission (λ_em_ = 449–520 nm, λ_ex_ = 405 nm) after 50 scans. By comparison, only 25% of the fluorescence intensity remained after six scans when the cells were stained with 50 nM cationic MT for 30 min (λ_em_ = 581–688 nm, λ_ex_ = 560 nm). The mitochondria have a large membrane potential (Δψ_m_ = −180 mV) responsible for generating ATP by oxidative phosphorylation. When the HeLa cells (mitochondrial region) were treated with 10 μM carbonyl cyanide *m*-chlorophenylhydrazone (CCCP), which causes the acidification of mitochondria, the mitochondrial Δψ_m_ decreased. The fluorescence intensity from CCCP treated cells stained with 50 nM cationic MT for 15 min decreased. This decrease demonstrates the inability of the dye to accumulate around the mitochondria when Δψ_m_ is reduced across the mitochondria (as shown in Figure 6b; λ_ex_ = 540–580 nm). Surprisingly, CCCP-treated cells stained with 5 μM TPE-TPP for 30 min and then excited at 330–385 nm retained their specificity to mitochondria (Figure 6c). The lipophilicity and di-cationic nature of TPE-TPP make it very sensitive for mitochondrial imaging, even when the Δψ_m_ of the mitochondria is reduced. 

#### 2.1.2. Gelator Emits in Solution/Sol States but Is Quenched in the Gelled State

Aggregation caused quenching is the opposite of AIEE. Förster and Kasper reported aggregation caused quenching long before the discovery of AIEE. They observed that the fluorescence intensity from pyrene decreased as its concentration was increased [59]. Many fluorophores such as pyrene, with an aromatic π-conjugated structure, are highly luminescent. Their aromatic structures make them hydrophobic and promote the formation of π-stacked aggregates in polar, H-bonding media, such as water. Stacking leads to the quenching of the monomer fluorescence but can initiate new forms of emission from excited dimers (i.e., excimers) [60]. The monomer quenching phenomenon limits the applications of many of these aromatic molecules in fields such as bio-imaging and drug delivery [61]. 

**Figure 7:**
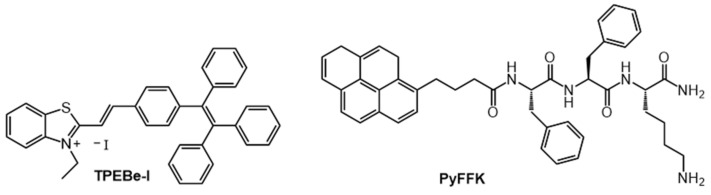
Molecular structures of TPEBe-I and PyFFK.

Zhao et al., developed a strategy to change the behavior of TPE-functionalized benzothiazolium salts with an iodide ion (TPEBe-I) from exhibiting aggregation caused quenching behavior to AIEE behavior by adding Hg^2+^ ions (which form HgI_2_) and reduce heavy atom quenching by iodide (Figure 7) [62]. Thus, aggregates of 20 μM TPEBe-I in 20 mM HEPES aqueous buffer (pH 7.4, with 1% DMSO) were weakly emissive when excited at 480 nm. However, strong and intense fluorescence was observed at ~650 nm upon the addition of 2 mM of Hg^2+^. 

**Figure 8:**
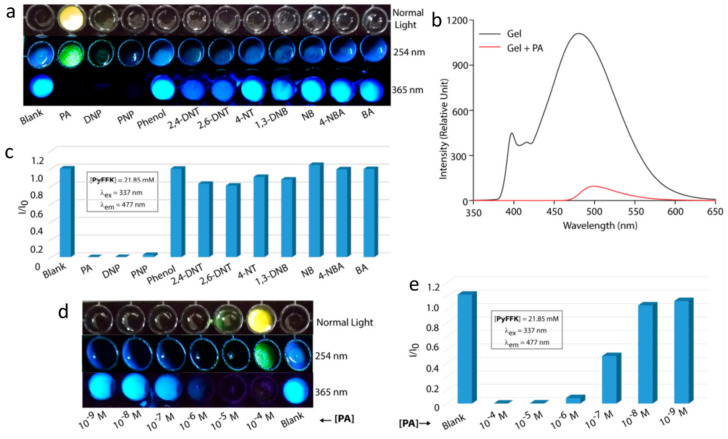
(**a**) Photographs of PyFFK gel-loaded wells of a multiwell plate in the presence or absence of different analytes under different lights. (**b**) Emission spectra of a gel sample in before and after treatment with picric acid (PA) solution (λ_ex_ = 337 nm). (**c**) The extent of quenching of the 477 nm band in the emission spectra of the gel samples in the presence of different analyte solutions. (**d**) Photographs of PyFFK gel-loaded wells of a multiwell plate after treatment with solutions of different concentrations of PA under different lighting conditions. (**e**) Extent of quenching of the emission at 477 nm for samples in (**d**). All experiments were performed at room temperature. Reprinted (adapted) with permission from [63]. Copyright (2019) American Chemical Society.

Pramanik et al., utilized aggregation-caused quenching to detect picric acid, a common component of explosives [63]. Their fluorescent gelator is based on a pyrenyl-functionalized peptide (PyFFK, Figure 7). Both solutions and gels of PyFFK in 1:1 water:acetonitrile showed aggregation-caused quenching in the presence of picric acid (Figure 8a–c). Thus, 95% of the monomeric pyrenyl emission (λ_em_ = 376 nm, λ_ex_ = 337 nm) of a 1 µM PyFFK solution was quenched in the presence of 10 equivalents of picric acid (K_sv_ of 3.94 × 10^−4^ M^−1^). However, addition of 30 µL of 1 mM picric acid to a 200 µL gel of 21.85 mM PyFFK led to 95% quenching of the pyrenyl excimer emission at 477 nm (λ_ex_ = 337 nm); the detection limit of PA was 10^–7^ M (Figure 8d,e). The same authors designed a paper-coated gel strip for contact mode detection of picric acid in vapor, aqueous and solid phases. 

### 2.2. Extrinsic (in Which Fluorescent Molecules/Dyes Have Been Added to the Gel System) and INTRINSIC (in Which the Gelator Itself Fluoresces in a Gelled State) Luminescent Gels

#### 2.2.1. Addition of Fluorescent Molecules/Dyes to the Gel System Are Required to Make the Resultant Gel Fluorescent

Examples of such systems include polymeric gels or supramolecular gels to which have been added fluorescent cross-linkers, fluorescent dyes, host–guest mediating metal ions, carbon dots or quantum dots. Each of these can impart luminescent properties to the gel.

**Figure 9:**
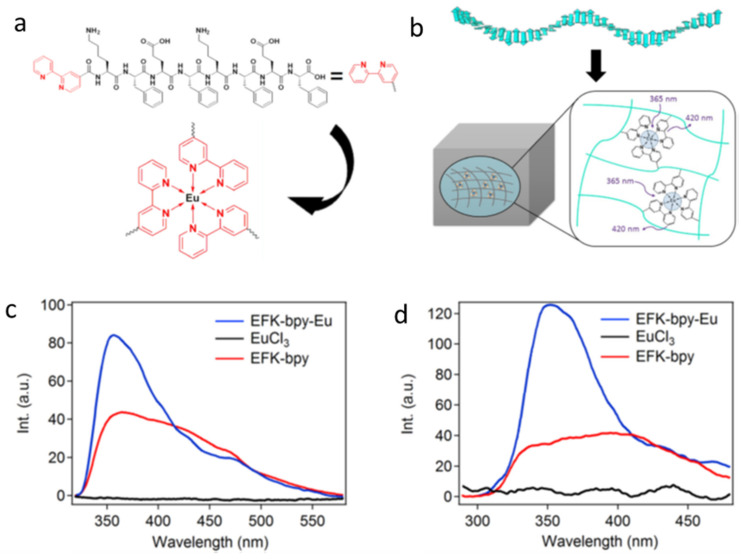
(**a**) Molecular structures of the EFK-bpy peptide and the structures of the EFK-bpy-Eu peptide. (**b**) Proposed schematic representation of the co-assembled hydrogel and the energy-transfer process. (**c**) The fluorescence emission spectrum of the EFK-bpy hydrogel (λ_ex_ = 300 nm; 4 mM), EuCl_3_ (λ_ex_ = 300 nm; 1.33 mM) solution, and EFK-bpy-Eu hydrogel (λ_ex_ = 300 nm; 1.33 mM). (**d**) The fluorescence emission spectrum of the EFK-bpy hydrogel (λ_ex_ = 254 nm; 4 mM), EuCl_3_ (λ_ex_ = 254 nm; 1.33 mM) solution, and EFK-bpy-Eu hydrogel (λ_ex_ = 254 nm; 1.33 mM). Republished with permission of Springer Nature, from [65].

Quite often, fluorophores are simply added to a non-emissive gel/sol to induce luminescence. The lack of covalent or non-covalent interactions between fluorophores and the gel matrix results in the aggregation of fluorophores or micro-separation of the fluorophores from the gel matrices, leading to imprecise or irreproducible optical properties [64]. To overcome such drawbacks, Xia et al., reported a fluorescent hydrogel with a peptide gelator (EFK-bpy) terminus capped with 2,2′-bipyridine (for metal chelation), which allows for complexation with external fluorophores (Figure 9a) [65]. EFK-bpy forms a self-assembled network in phosphate saline buffer (pH 7) due to ionic and π–π stacking interactions. The addition of EuCl_3_ to EFK-bpy yields an octahedral complex (EFK-bpy-Eu) through metal-ligand coordination and imparts fluorescent properties to the system (Figure 9b). The hydrogel of 4 mM EFK-bpy was weakly fluorescent when excited at 300 nm, while the 1.33 mM EuCl_3_ showed no fluorescence. However, strong emission centered at 420 nm was observed from the 1.33 mM EFK-bpy-Eu hydrogel due to complexation (Figure 9c). The addition of EuCl_3_ not only forms a coordinate bond which prevents aggregation and the leaking of fluorophore (when not chemically bonded with gelators), but also increases, by six-fold, the storage modulus of the gel. 

#### 2.2.2. Gelator Fluoresces in Its Gel Phases

The emitting unit of most gelators of this type contains a π-conjugated, rigid, and planar backbone. Common examples of the emitting unit include cyclic, conjugated aromatic groups, such as substituted phenyls and naphthyls (especially, naphthalene diimides (NDIs)), acenes and TPEs. Although azobenzene groups themselves, show very little, if any, emission from their excited states, they are useful moderators of emission from other groups within an LMOG because of their ability to undergo *cis-trans* isomerizations, which can change the overall molecular shape and, thus, the ability of the LMOGs to become emissive [66]. Less common are acyclic gelators, such as molecules with α-dicarbonyl groups.

**Figure 10:**
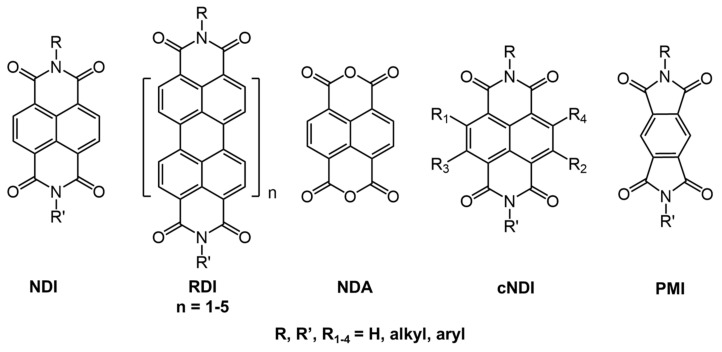
Structure of naphthalene diimide (NDI) and derivatives of it with potential photophysical, self-assembly, and functional properties. Reprinted (adapted) with permission from [67]. Copyright (2016) American Chemical Society.

NDI-based systems (Figure 10) have been studied extensively due to their ability to act as n-type semiconductors [67], ability to accept π-electrons [68], and their variable absorption and emission properties through functionalization of the nitrogen atoms [69]. 

**Figure 11:**
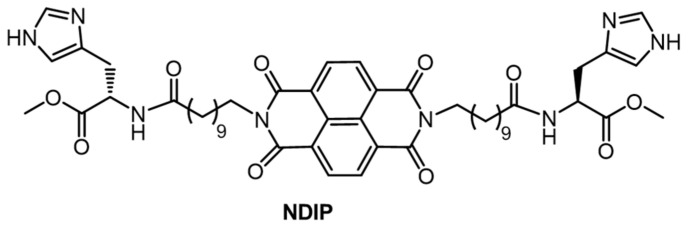
Molecular structure of NDIP.

For example, Gayen et al., studied gel systems based on NDI derivatized with a histidine-containing peptide (NDIP, Figure 11) [32]. NDIP behaves as a bola amphiphile (i.e., both an organogelator and a hydrogelator), having an NDI core and methylene units at the center and two imidazole units at the termini. During the self-assembly of the NDIP and the subsequent formation of the gelator network, in aqueous phosphate buffer (pH 7.46, CGC = 0.14% *w*:*v*) and in toluene (CGC = 0.12% *w*:*v*), the presence of peptides at the chain termini enhances H-bonding, and the long alkyl chains introduce specific van der Waals and hydrophobic interactions. Additionally, the presence of π–π interactions from the NDI core enhances aggregation as well as produces a strong blue-fluorescent emission in the aggregated state, which is otherwise absent in the monomeric state. The excited singlet state of NDIP dissolved in hexafluoro-2-propanol has a short lifetime, 0.078 ns (λ_ex_ = 340 nm, λ_em_ = 420–460 nm) compared to the hydrogel and organogel (in toluene) which have bi-exponential decays with longer average lifetimes of 1.17 and 1.01 ns, respectively. 

**Figure 12:**
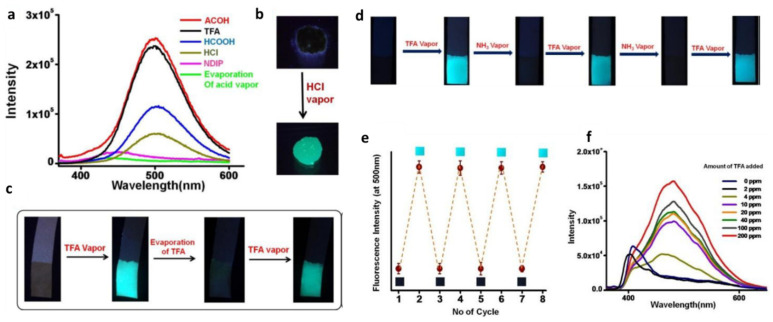
(**a**) Fluorescence spectra of NDIP xerogels upon exposure to different volatile acid vapors. (**b**) Fluorescence "turn on" of NDIP xerogels upon exposure to HCl vapor. (**c**) Fluorescence on/off behavior by exposure and evaporation of acid vapor using a paper strip impregnated with NDIP. (**d**) Fluorescence on/off switching upon exposure to TFA and ammonia vapors of the paper strip device impregnated with NDIP. (**e**) Fluorescence on/off cycle of NDIP in the presence of acid and base vapors. (**f**) Fluorescence spectra of NDIP in milli-Q water in different amounts (ppm) of trifluoroacetic acid (TFA). From [32]. Copyright Wiley-VCH GmbH. Reproduced with permission.

Furthermore, aggregated NDIP can detect the presence of acids. The non-fluorescent xerogel state becomes emissive (λ_em_ =~500 nm, λ_ex_ = 365 nm) in the presence of acid vapors, including formic acid, acetic acid, trifluoroacetic acid (TFA), HCl, HNO_3_ and H_2_SO_4_ (Figure 12a–c). Because the fluorescence emission disappears in the presence of NH_3_ vapors, NDIP is a fluorescent switch for detecting acid and base vapors (Figure 12d–f). The average lifetime of excited NDIP in the aggregated state in the presence of formic acid, 9.07 ns (λ_ex_ = 340 nm and λ_em_ = 480–500 nm), is much longer than that of the native gel.

**Figure 13:**
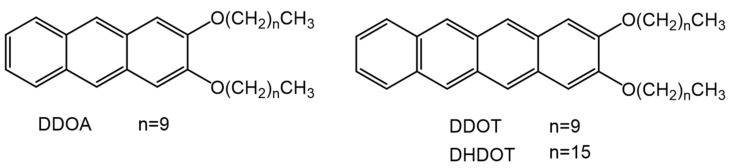
Molecular structures of DDOA, DDOT and DHDOT.

Linear acenes, such as anthracene, tetracene and pentacene, have been studied extensively due to their excellent optical properties and charge mobilities, and are often utilized in the field of photonics, photovoltaics, light harvesting and optoelectronics [70,71,72]. Their low solubilities in many solvents, however, limits their applicability. To improve solubilities, Brotin et al., substituted the anthracenyl group with linear, long-chain alkoxy units at the 2 and 3 positions [73]. Among various substituted anthracenes, 2,3-didecyloxyanthracene (DDOA, Figure 13) not only showed improved solubilities but was able to gelate a variety of aliphatic alcohols and amines at low concentrations due to van der Waals, π–π stacking and induced dipole–dipole interactions. Thus, 6 × 10^−5^ M DDOA in methanol became a gel below −42 °C and showed a red shift in fluorescence emission compared to the sol (above −31 °C) when excited at 365 nm. Desvergne et al., also studied tetracene derivatives with long alkoxy chains, 2,3-didecyltetracene (DDOT) and 2,3-dihexadecyltetracene (DHDOT) (Figure 13) [74]. While both are capable of gelating aliphatic alcohols, DHDOT showed a lower CGC and even gelled linear alkanes. The authors attribute this difference to the balance between the sizes of the fused aromatic rings and the linear chains that aid self-assembly.

**Figure 14:**
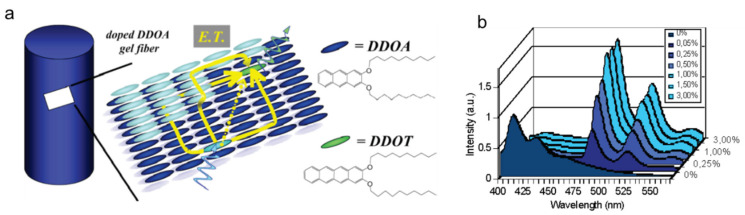
(**a**) Simplified representation of a doped DDOA gel fiber (enlargement of three layers of sheets of DDOA) is represented, one excited donor (light blue) and one emissive acceptor (green), as well as energy transfer pathways (E.T., yellow arrows) for the direct process (dotted arrow) and several possibilities for exciton migration. Right: Chemical structures of DDOA and DDOT (**b**) Emission spectra of DDOA (2.0 × 10^–3^ M) gels in DMSO at 293 K, λ_ex_ = 384 nm with increasing amounts of added DDOT (mol %). Reprinted (adapted) with permission from [76]. Copyright (2005) American Chemical Society.

Additionally, Desvergne et al., investigated the light harvesting properties of mixed gels to observe electronic energy transfer between two gelators containing a different type of acene (Figure 14a) [74,75]. In that regard, 2 × 10^−5^ M DDOA in methylcyclohexane, when doped with 0.75% DDOT, showed same fluorescence emission as neat 2 × 10^−5^ M DDOA in the solution phase of methylcyclohexane. However, upon gelation of the doped solution at low temperature (−103 °C), the emission characteristics of the tetracenyl group of DDOT changed appreciably (λ_ex_ = 366 nm). In the doped system, the authors followed light harvesting from the anthryl donor unit to the tetracenyl acceptor unit. Upon varying the amount of DDOT (0–3%) in 2 × 10^−5^ M DDOA in DMSO, a change in the emission intensity was observed (Figure 14b, λ_ex_ = 384 nm), and the maximum emission intensity was observed with 1% doping.

## 3. Syntheses 

A common strategy to design a luminescent LMOG is to incorporate a fluorescent or phosphorescent group within the gelator structure. Here, we discuss a few synthetic routes for obtaining LMOGs involving ᴫ-conjugated and acyclic systems. The development of general synthetic schemes for classes of molecules derived from a known LMOG are important because small modifications within an LMOG structure frequently alter its gelating abilities and gel properties.

**Figure 15:**
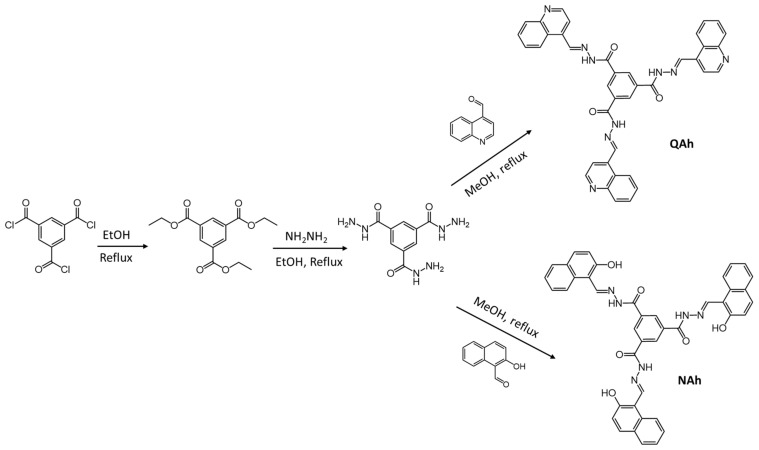
Syntheses of QAh and NAh. Republished from [77] with permission from the Royal Society of Chemistry.

For example, Sharma et al., synthesized molecules with an acylhydrazone substituent, QAh and NAh (Figure 15), which have a selective response to cyanide ions in the gel and solution states [77]. The 10 mg QAh was gelated in 1 mL of DMSO:water (1:1, *v*:*v*) due to the H-bonding ability of the quinoline unit. At same concentration, NAh did not form a gel even when the DMSO:water ratio was changed. This difference was ascribed to the intramolecular H-bonding ability of NAh (N.B., the hydroxyl group at the *ortho* position). 

QAh showed AIEE behavior in different water:DMSO mixtures; the maximum emission intensity was found in 90:10 (*v*:*v*) water:DMSO, while no emission was observed in neat DMSO. Furthermore, the addition of 15 equiv. of CN^−^ ions in 10 µM QAh solution in DMSO caused a red-shift in absorbance, from ~335 to ~416 nm (where it was bright yellow). Additionally, the QAh detected CN^−^ selectively (detection limit of 1.5 µM) in the presence of other anions (such as sulphate, nitrate, phosphate, halides, carboxylate, perchlorate and thiocyanate). Upon the addition of CN^−^ ions to the white gel of QAh in 1:1 (*v*:*v*) DMSO:water, a yellow precipitate formed as a result of the disruption of the gel network. Sharma et al., used this change in an experiment in which a cotton swab was dipped in the gel in the presence of CN^−^ ions, causing the appearance to go from white to orange.

**Figure 16:**
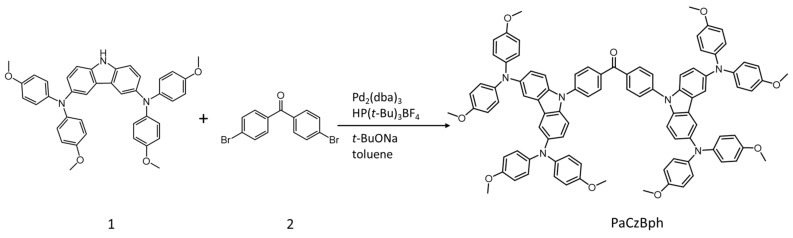
Synthesis of PaCzBph. Reprinted from [78] with permission from Elsevier.

Shan et al., made a butterfly-shaped, π-conjugated, LMOG, PaCzBph, via a Buchwald–Hartwig reaction using *N*^3^,*N*^3^,*N*^6^,*N*^6^-tetrakis(4-methoxyphenyl)-9*H*-carbazole-3,6-diamine (1) and bis(4-bromophenyl)methanone (2) (Figure 16) [78]. The ability of PaCzBph to gelate a range of organic solvents was attributed to π–π stacking along the molecular backbone and to dipole–dipole interactions between the carbonyl group and terminal methoxy groups; the CGC was 1.7 mg/mL for PaCzBph in acetone. The absorption band of 3 μM PaCzBph in THF at 305 nm arises from a π–π* transition and at 400 nm due to intramolecular charge transfer from the carbazole to benzophenone moieties. The band undergoes a slight red shift on formation of organogels and is another example of AIEE. The corresponding solution state exhibited weak fluorescence (φ_F_ < 0.1%) when excited at 400 nm. By contrast, the gel emitted a bright orange emission (φ_F_ = 10%).

**Figure 17:**
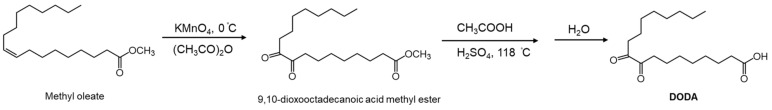
Synthesis of DODA [44]. Copyright John Wiley and Sons. Reproduced with permission.

Fluorescent and phosphorescent LMOGs producing gels with AIEE properties need not contain π-conjugated backbones, metals, or heavy atoms. For example, Zhang et al., exploited the known ability of α-diketo groups to both fluoresce and phosphoresce in solution at room temperature [79,80] to construct a structurally simple LMOG, DODA [44]. The synthesis of DODA involved oxidation by potassium permanganate at the 9 and 10 positions of methyl oleate to yield 9,10-dioxooctadecanoic acid methyl ester, followed by hydrolysis (Figure 17). The long alkyl chain promotes van der Waals interactions within the gel network and the carboxylic acid group at the terminal position enhances self-assembly via end-on H-bonding. The α-diketo group along the chain adds some dipole–dipole interactions. The morphologies of the DODA gel assembles and the photoluminescent properties of their gels and sols will be discussed in Section 5. 

**Figure 18:**
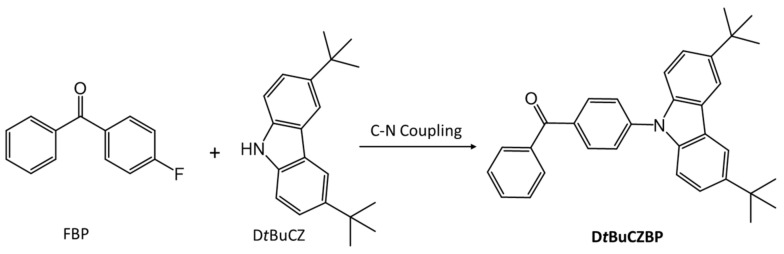
Synopsis of the synthetic route to DtBuCZBP.

Another example of an LMOG that is both fluorescent and phosphorescent is 4-(3,6-di-*tert*-butyl-9*H*-carbazol-9-yl)] benzophenone, D*t*BuCZBP [81]. It was synthesized by C-N coupling between 4-fluorobenzophenone (FBP) and 3,6-di-*tert*-butyl-9*H*-carbazole (D*t*BuCZ) (Figure 18). D*t*BuCZBP forms an opaque gel in DMSO at concentrations >20 mg/mL. Under irradiation at 365 nm, neat D*t*BuCZBP emits blue light centered at 433 nm. Sols of 30 mg/mL D*t*BuCZBP in DMSO (presumably by heating the gel) emit yellow light centered at 539 nm. The blue-white emission from the gel, at the same concentration, consists of bands centered at 460 nm with a shoulder at 485 nm. The emissions indicate changes in the packing arrangements in the different phases. Furthermore, 0.05 ms delayed emission studies at room temperature for neat D*t*BuCZBP showed dual emissive behavior, with delayed fluorescence centered at 436 nm and phosphorescence centered at 493 nm. No dual fluorescence–phosphorescence emission was observed for D*t*BuCZBP that had been mechanically powdered or was in solution. 

## 4. Properties of Luminescent Gels

In this section, we discuss some properties of gel networks involving LMOGs with different functional groups and, especially, the effect of changing alkyl chain lengths. Additionally, discussed are changes in the luminescent properties of LMOGs upon gel formation and their responses to external stimuli. In this regard, we consider and compare the absorption and emission characteristics, quantum efficiencies and decay lifetimes of the LMOGs in their gels.

**Figure 19:**
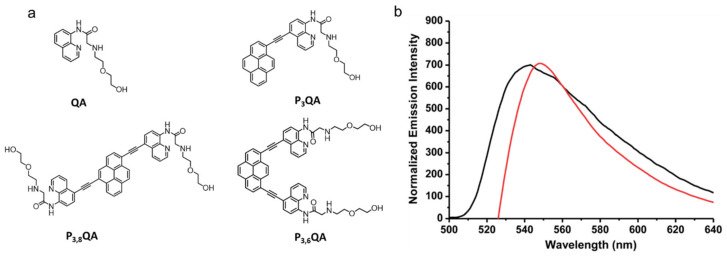
(**a**) Molecular structures of QA and ethynylpyrenyl derivatives of QA (P_3_QA, P_3,8_QA and P_3,6_QA). (**b**) Normalized fluorescence emission spectra of P_3,6_QA in the gel state (CGC concentration) in the absence of Zn^2+^ (red curve) and in the presence of 0.5 equiv. of Zn^2+^ (black curve) in acetone. Republished from [28] with permission of The Royal Society of Chemistry.

The fluorescence of solutions of 2-((2-(2-hydroxyethoxy)ethyl)amino)-N-(quinolin-8-yl)acetamide (QA, Figure 19a) changes upon binding to Zn^2+^ [82]. Huang et al., used this approach to design derivatives of compound QA with ethynylpyrenyl groups (Figure 19a) that form gels and are useful for sensing Zn^2+^ ions [28]. However, QA, P_3_QA and P_3,8_QA were unsuccessful in forming gels; only P_3,6_QA was able to gelate a variety of high and low polarity organic solvents as well as mixtures of water and organic solvents. The presence of pyrenyl as well as quinoline units enhances π–π stacking interactions. The CGCs were reported to be ~8.2 mg/mL in acetone, chloroform, and dichloromethane and ~13.7 mg/mL in dioxane, tetrahydrofuran and ethyl acetate. The self-assembly and thermo-reversibility of gels formed from P_3,6_QA were attributed to the amide and NH groups, which stabilize the gel network through H-bonding while also acting as Zn^2+^ acceptor sites. In acetone solutions, emission from 10 μM P_3,6_QA is centered at 468 and 495 nm while in the gelated state, pairs of pyrenyl units emit as a broad band centered at 546 nm; this emission is indicative of excimer formation. 

The specific sensitivity of the gel towards Zn^2+^ ions was also observed (Figure 19b). For example, addition of 0.1 equiv. of Zn^2+^ to a P_3,6_QA in acetone gel led to a partial gel-to-sol transition after 10 min while addition of 0.5 equiv. of Zn^2+^ resulted in a complete gel-to-sol transition within 30 s. The transition is attributed to disruption of the H-bonding network. The fluorescence emission band of the sol was blue shifted (from 546 nm in the gel to 539 nm in the sol). 

**Figure 20:**
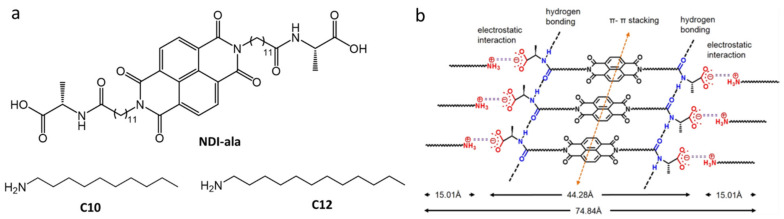
(**a**) Molecular structures of NDI-ala and amines, *n*-decylamine (C10) or *n*-dodecylamine (C12). (**b**) Tentative model (based on data from Fourier transform infrared (FTIR), PXRD, and SAXS) for the molecular packing arrangement of the two-component co-assembled system, NDI-ala12. The molecular length of C12 in its extended conformation was used to estimate the total length of NDI-ala12. Reprinted (adapted) with permission from [84]. Copyright (2016) American Chemical Society.

Aggregation-caused quenching is commonly found when π-conjugated molecules are dispersed in water [83]. Nandi et al., utilized AIEE from hydrogels in phosphate buffer (pH 7.46) [84]. The gelator had two-components, a derivatized NDI-core substituted with the bola amphiphilic peptide (NDI-ala) and a long alkyl chain primary amine (Figure 20a). The NDI-ala alone was unable to form a gel in phosphate buffer. However, it formed gels, NDI-ala10 and NDI-ala12, when *n*-decylamine (C10) or *n*-dodecylamine (C12) was added in 1:2 NDI-ala:amine molar ratios. The CGC values were very low: 0.07% and 0.04% (*w*:*v*) for NDI-ala10 and NDI-ala12, respectively. The addition of an amine introduced attractive electrostatic interactions between the carboxylate ion in NDI-ala and ammonium ions produced on protonation of the amine. The amines increase the aggregating forces from non-covalent intermolecular interactions among NDI-ala (e.g., π–π stacking, H-bonding and van der Waals forces) (Figure 20b). However, gelation was not observed when secondary amines, tertiary amines, or aromatic amines were added. In its monomeric state, NDI-ala (0.5 mM) in THF exhibits very weak fluorescence centered at 410 nm (λ_ex_ = 340 nm); in phosphate buffer where it becomes aggregated, a strong intense greenish-yellow fluorescence, centered at 460 and 550 nm (λ_ex_ = 340 nm), was observed with φ_F_ = 3.6%. The addition of amines increased the quantum yields. For example, C12 in NDI-ala12 (0.5 mM) increased φ_F_ to 5.4% and gave an emission centered at 430 nm and another broad peak at 530–560 nm. Xerogels of NDI-ala10 and NDI-ala12 were found to be semi-conducting, with a conductivity of ~5.5–6 × 10^−6^ S cm^−1^. This observation opens the possibility of using the xerogels in optoelectronic devices.

**Figure 21:**
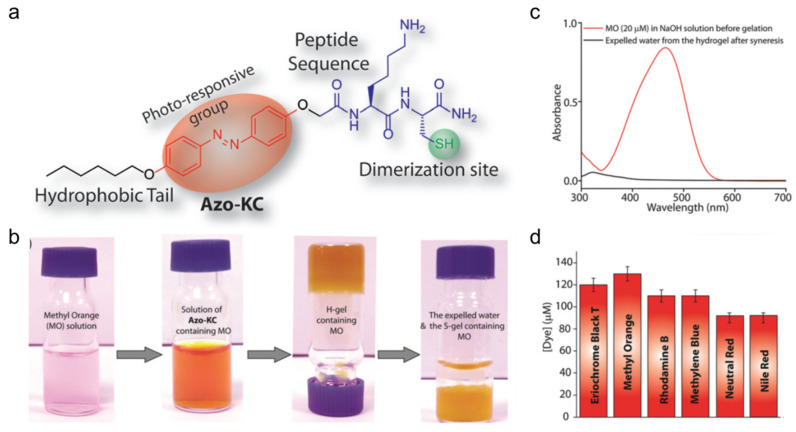
(**a**) Molecular structure of Azo-KC. (**b**) Photographs showing methyl orange removal from the H-gel upon irradiation at 365 nm and induced syneresis. (**c**) UV-vis spectra of methyl orange and the expelled water from (**b**). (**d**) Maximum concentrations of different dyes, which can be removed completely during syneresis of the hydrogel (1.15%). All measurements were carried out at room temperature. Republished from [85] with permission of The Royal Society of Chemistry.

Das et al., investigated a hydrogelator containing azobenzenyl and cysteine-based peptide units, Azo-KC (Figure 21a), whose gels in water with small amounts of NaOH undergo synereses due to *trans*-to*-cis* isomerization of the azo group when irradiated in the UV region [85]. Thus, the Azo-KC hydrogel (H-gel) shrinks up to 50% of its original volume (shrunken gel, S-gel) by expelling excess water when irradiated in the UV region. The critical gelator concentration for the H-gel form is 1.15 wt%, with a gel-to-sol transition temperature of 57 °C. Irradiation with visible radiation at 420 nm, that causes *cis*-to*-trans* isomerization, does not lead to reformation of the H-gel. No syneresis was detected on incubating the H-gel at room temperature for several days, and only slight changes were observed on heating the gel at 45 °C. Apart from non-covalent interactions, the self-assembly is assisted by disulfide linkages. The H-gel was insoluble in water and aqueous buffers (pH 1–13) but dissolved when exposed to disulfide bond-breaking agents. FESEM images of the H-gel show thin (2–5 nm diameter) fibers that become long, rod-like structures (~1μm) after UV irradiation at 365 nm. At the CGC, the absorption maximum of the H-gel, corresponding to the π–π* transition of a *trans* azobenzenyl group, is centered at ~342 nm. The maximum of the S-gel is shifted hypsochromically to 321 nm, and a new peak at 445 nm (n-π* transition of *cis*-azobenzenyl) appears.

Additional experiments suggest potential applications for the H-gel as an agent to entrap small, toxic molecules. Thus, the H-gels prepared in the presence of dyes (shown in Figure 21d), showed no free dye molecules in the water parts by UV-vis absorption spectroscopy even after UV irradiation (i.e., syneresis) (Figure 21b,c). For example, at 1.15 wt%, the H-gel successfully entrapped 120 μM of methyl orange (Figure 21d). 

**Figure 22:**
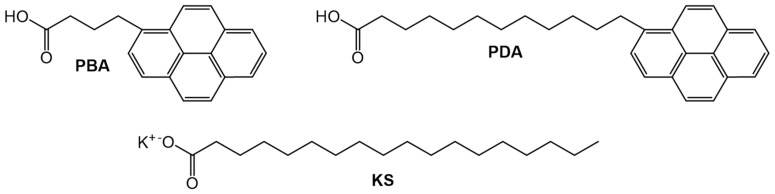
Molecular structures of PBA, PDA and KS.

Jenkins et al., used static and dynamic fluorescence to study the packing arrangements within the gel networks of model bilayers of potassium stearate (KS) using ω-(1-pyrenyl)alkanoic acids (PBA and PDA, Figure 22) with different chain lengths as the probes [86]. These amphiphilic gels containing stearate anions arranged in cylindrical smectic-like arrangements [87,88,89]. The alkyl chains of the gelator reside in the interior of the layers and the polar carboxylate groups are at the aqueous interface. In their 10^−5^ M solution phases in tridecane, the fluorescence lifetime of PBA (τ_F_ = 272.4 ns) is 80 ns longer than that of PDA (τ_F_ = 192.4 ns). While the 10^−5^ M PDA:KS (1:1, w:w) hydrogel has τ_F_ = 199.2 ns, greater than that of the corresponding PBA:KS hydrogel (τ_F_ = 129.9 ns). The authors ascribed these increases in the fluorescence lifetimes of PDA in the gel phase to changes in the lowered proximity of the pyrenyl group to the aqueous potassium ion quenchers. Furthermore, comparison of intensity ratios of the first and third vibronic emission bands (*I*_1_/*I*_3_) from the monomeric manifold of the pyrenyl groups at 374 ± 2 and 385 ± 2 nm, respectively, supports the contention that there is greater exposure of pyrenyl groups in PBA than in PDA to the polar region of the phase. 

## 5. Morphology and Packing of Fluorescent Gels

Luminescent properties depend on both the morphology of the gels and the position of the functional groups attached to the gelator. Zhang et al., studied the morphologies and photophysical properties of gels formed from DODA (Figure 17) [44]. At room temperature, a 6.0 × 10^−3^ mol L^−1^ solution in dry THF had its maximal emission at 481 nm with a shoulder at 510 nm (λ_ex_ = 425 nm). At, −15 °C, a degassed solution showed an additional emission peak centered at 548 nm that was attributed to phosphorescence [79].

**Figure 23:**
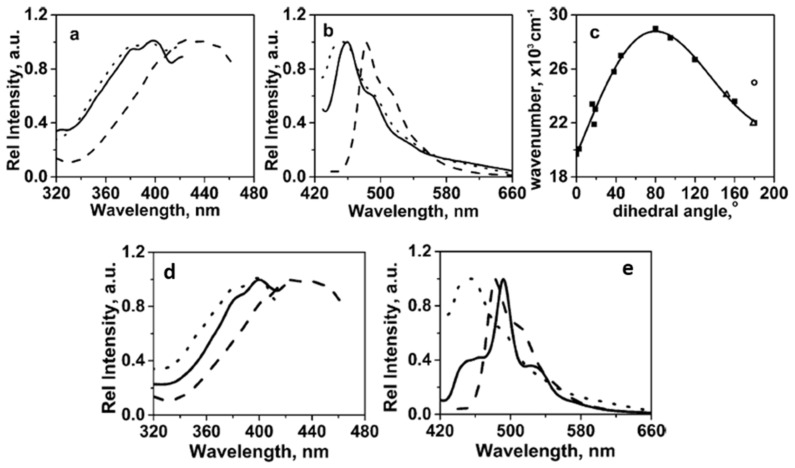
(**a**) Excitation and (**b**) emission spectra of a 5 wt% DODA in 1-octanol gel at 25 °C (solid line, λ_em_ = 458 nm, λ_ex_ = 400 nm), sol at 45 °C (dashed line, λ_em_ = 481 nm, λ_ex_ = 425 nm), and melt-solidified DODA at 25 °C (dotted line, λ_em_ = 458 nm, λ_ex_ = 400 nm). (**c**) Dependence of the excitation wavenumber maxima on the inter-carbonyl dihedral angle of a series of α-diketo containing molecules at 25 °C in solution (solid squares; republished from [90] with permission of The Royal Society of Chemistry); a 5 wt% DODA in 1-octanol gel at 25 °C (open circle); a 5 wt % DODA in 1-octanol sol at 45 °C (open triangle). The range of multiple excitation maxima of the 5 wt % DODA in 1-octanol sol is indicated by the curve between the two open triangles. (**d**) excitation, and (**e**) emission spectra at 25 °C of gels of 5 wt % DODA in 1-octanol after incubating the sols at 0 °C (dotted line, λ_em_ = 458 nm, λ_ex_ = 400 nm), 30 °C (solid line, λ_em_ = 458 nm, λ_ex_ = 400 nm), and 45 °C (dashed line, λ_em_ = 481 nm, λ_ex_ = 425 nm). Reproduced with permission from [44]. Copyright John Wiley and Sons.

The change in dihedral angle between the carbonyl groups of an α-diketone is known to change the position of the absorption band; a red-shift is observed as the dihedral angle increases from 90 to 180° [90]. The excitation and emission peaks of 5 wt % DODA in 1-octanol show a marked blue shift (~25 nm) as the sol passes into the gel state (Figure 23a,b). From Figure 23c, the calculated dihedral angle between the two carbonyl groups of DODA in 1-octanol in its sol are in the range of 155–180°. However, an anti-conformation (i.e., an angle near 180°) is not attained in the gel state due to the packing preference in the aggregate. As a result of additional dipole–dipole interactions, which are indicated by X-ray diffraction data, the value of the angle in the gel state cannot be calculated from the spectral maximum. Moreover, the morphologies of the DODA objects in the 1-octanol gels differ when prepared at different incubation temperatures. For example, the gel objects prepared by incubating at 0 °C are small fibers which become longer and thicker when the incubation temperature is 30 °C, and appear to be platelets (co-existing with a few fibers) at 35 °C. These differences in morphology resulted in significantly different fluorescence emission spectra (Figure 23d,e).

**Figure 24:**
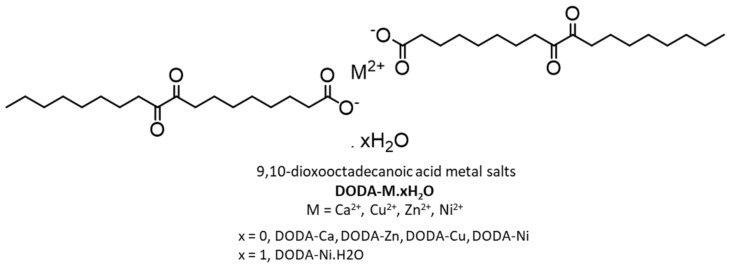
Structures of salts examined as metallo gelators derived from DODA. Republished from [91] with permission of The Royal Society of Chemistry.

Zhang et al., conducted additional studies using Ca, Ni, Cu, Zn salts of DODA; all metals in their +2-oxidation state (Figure 24) [91]. The salts DODA-Cu and DODA-Ni·H_2_O formed gels in a range of organic liquids. In one solvent, the CGC was lower, and the T_g_ and yield strain values were higher for the gels of DODA-Cu and DODA-Ni·H_2_O than those of DODA. These results indicate stronger aggregation for the salt gelators due to additional electrostatic interactions and better thermal mechanical stability. In 1-octanol at 5 wt %, DODA-Cu/DODA-Ni·H_2_O gels do not show a blue shift in their emission spectra like that found in excitation spectra for the DODA gels when sols became gels. The comparable photophysical properties of the gel and sol state of 5 wt % DODA-Cu/DODA-Ni·H_2_O gels in 1-octanol suggests a lack of dipole–dipole interactions between α-diketo groups in metal-salts of DODA such as that mentioned in the previous paragraph. 

**Figure 25:**
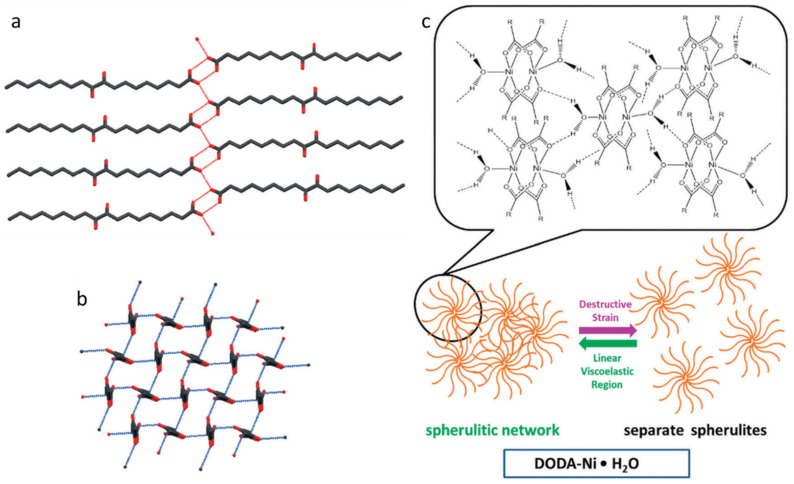
(**a**) 2D packing diagram of DODA viewed perpendicular to the polymethylene chains; the red dashed lines indicate the positions of H-bonds; (**b**) a 2D packing diagram of DODA viewed along the axis of the polymethylene chains; the blue dashed lines indicate dipole–dipole interactions. Reproduced from [44] with permission. Copyright John Wiley and Sons. (**c**) Schematic representation of proposed molecular packing arrangements and proposed mechanisms for mechano-destruction and reformation of self-assembled fibrillar networks in the DODA-Ni·H_2_O gels. Republished from [91] with permission of The Royal Society of Chemistry.

Single crystals of DODA (Figure 25a,b) indicate an attractive dipole–dipole interaction with a short intermolecular C and O distance of 2.9 Å (the sum of the van der Waals radii is 3.2 Å). Although the single crystal structure for DODA-Ni·H_2_O was not obtained, a pseudo square planar with an intermolecular C and O distance of 4.4 Å (Figure 25c) was proposed based on the crystal structure of nickel(II) propanoate monohydrate and the WAXS profile of neat DODA-Ni·H_2_O (*q* = 1.5 Å^−1^ ). Thus, the large intermolecular C-O distance results in a lack of dipole–dipole interactions.

**Figure 26:**
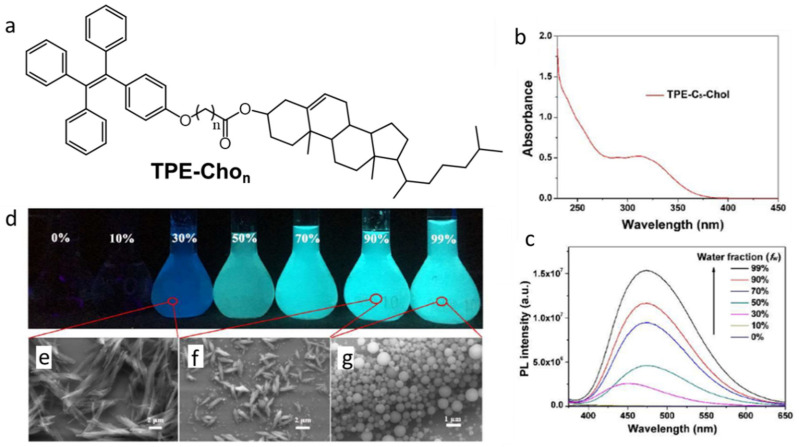
(**a**) Structure of TPE-Cho_n_ (n = 1,4,5,6). (**b**) UV−vis absorption spectrum of 1 × 10^−4^ M TPE-Cho_5_ in dichloromethane. (**c**) Emission spectra (λ_ex_ = 355 nm) of 1 × 10^−4^ M TPE-Cho_5_ in different acetone:water mixtures. (**d**) Images under 365 nm radiation of TPE-Cho_5_ in acetone:water mixtures, with different water fractions, and SEM images of the aggregates at water contents equal to (**e**) 30%, (**f**) 90% and (**g**) 99%. Reproduced from [92] with permission. Copyright Wiley-VCH GmbH.

Chen et al., reported AIEE from gelators containing cholesterol and TPE substituents with O(CH_2_)_n_COO- linkers, TPE-Cho_n_ (Figure 26a) [92]. TPE-Cho_n_ uses π-π stacking and steroidal interactions to form aggregates that form gels with acetone and DMF. The SEM images of TPE-Cho_1_ and TPE-Cho_5_ xerogels from acetone show long ribbon-like structures with ~200 nm thicknesses. The TPE-Cho_4_ and TPE-Cho_6_ (i.e., with even number of methylene units in the spacer) form sheet-like structures with thicknesses of ~100 nm. The sol of TPE-Cho_5_ (40 mg/mL) in acetone is less luminescent than when cooled to its gel state. Because the sample is thermo-reversible (with ultrasonication), its emission intensity can be increased and decreased repeatedly in a cyclic fashion as it is cooled to below its gelation temperature and then heated to a temperature above it. Moreover, 10^−4^ M TPE-Cho_5_ in a 70:30 acetone:water mixture, which emits blue fluorescence when excited at 355 nm, undergoes a red shift as the water fraction is increased (Figure 26b–g). The shift was attributed to changes in the packing of the aggregates: in 30% water, rod-like crystallites with a thickness ~2 μm were present; in 99% water, spherical nanoparticles with diameters <1 μm were observed. Moreover, TPE-Cho_n_ shows thermo- mechano-, vapo-chromic properties in the condensed phase. For example, ground solid TPE-Cho_5_ has an emission maximum of 473 nm whereas the unground solid has an emission maximum of 453 nm; passing hexane vapors over TPE-Cho_5_ improved its crystalline ordering and led to a blue shift in fluorescence.

**Figure 27:**
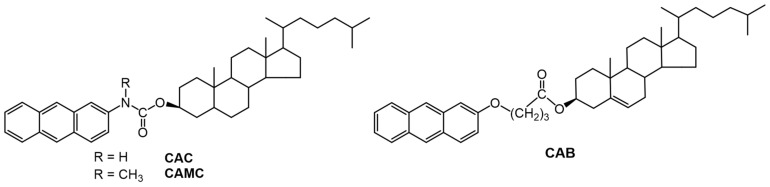
Molecular structures of CAC, CAMC and CAB.

Lu et al., studied LMOGs based on aromatic-linker-steroid (ALS) molecules, such as 5α-cholestan-3β-yl N-(2-anthryl)-carbamate (CAC) and 5α-cholestan-3β-yl N-(2-anthryl)-methylcarbamate (CAMC) (Figure 27) [93]. CAC successfully gelated a variety of alkanes and alcohols. However, its methyl derivative (CAMC) did not form gels with alkanes and formed weak gels with 1-butanol and 1-pentanol. While there is no clear evidence of intermolecular H-bonding interactions within the carbamates of the CAC molecules in their gels, the strong π–π and π---H-N interactions contribute to their stability. X-ray analysis of a single crystal of CAMC showed the presence of C=O---H_3_C-N type interactions (which aid gelation) but no clear π–π interactions. 

The absorption spectra of a 0.3 wt% CAC gel in 1-pentanol showed a band at 420 nm (not present in the sol phase) and differences between the intensities of a band at 395 nm in the gel and sol phases. The authors assigned these bands to co-existing aggregates in the gelated phase. In the gel, the formation of strands of CAC molecules leads to altered intensity of 395 nm band and the presence of junction zones (cross-linking points in the gel network) are responsible for a band at 420 nm. By contrast, no aggregates were discernible in the absorption spectra of 10^−2^ wt% CAMC in 1-pentanol solution (λ_max_ = 380 nm), although a broad peak centered at 410 nm was apparent when the concentration was increased to 1 wt%. 

Furman et al., studied the kinetics of aggregation of an ALS gelator, cholesteryl-4-(2-anthryloxy)butanoate (CAB, Figure 27) by fluorescence spectroscopy. The morphology of the gel networks of this gelator is sensitive to the rate of cooling from the sol phase, concentration of the gelator, and solvent characteristics [94]. Thus, 1.5 wt% CAB gels in different compositions of 1-octanol (up to 75 wt%) in hexadecane showed an emission maximum at 422 nm (λ_ex_ = 346 nm) that is characteristic of CAB in hexadecane gels. However, at 80–85 wt% 1-octanol in hexadecane, the emission maxima depended on the rate at which the sol was cooled to the gel phase. When a "fast-cooling" method (ca. 8° C/min) was employed, the emission maximum was observed at 422 nm; when a "slow-cooling" method (ca. 0.5° C/min) was used, the emission maximum was shifted to 427 nm (that is characteristic of CAB in 1-octanol gels). Above 89 wt% of 1-octanol in hexadecane, the CAB gel emitted at 427 nm, irrespective of the cooling method adopted. 

Apart from variable optical properties, the gels of CAB showed different morphologies and gel melting temperatures in different solvents. Lin et al., examined the morphologies of a ~2 wt% CAB gel in neat 1-octanol, using electron microscopy. The objects were spherulitic, having diameters of 6–8 μm; in hexadecane the diameters were much larger, ca. 200 μm [17].

**Figure 28:**
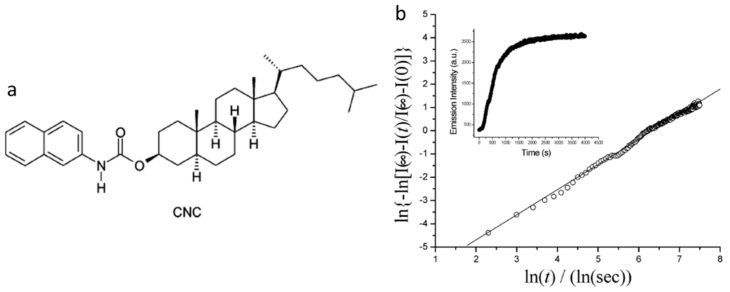
(**a**) Molecular structure of CNC; (**b**) Avrami plot of fluorescence data according to the Avrami equation (slope = 1.08 (R^2^ = 1.00)). Inset: Plot of emission intensity (λ_em_ = 375 nm; λ_ex_ = 318 nm) from a 1.0 wt % CNC in n-octane sample incubated at 1.1 °C. Reprinted (adapted) with permission from [95]. Copyright (2005) American Chemical Society.

In another example based on ALS molecules, Huang et al., studied the kinetics of gel formation of 5α-cholestan-3β-yl N-(2-naphthyl)-carbamate (CNC, Figure 28a) in n-octane and in n-dodecane, using techniques such as circular dichroism, fluorescence, small angle neutron scattering and rheology, and analyzing the results according to different kinetic and structural models [95,96]. Both the concentration of CNC in n-octane (from 0.89 wt % to 3.0 wt %) and temperature (from 1.1 °C to 39.2 °C) were varied. In one study, the excitation and emission spectra in 1.0 wt % CNC in n-octane gel (λ_ex_ = 333 nm and λ_ex_ = 357 nm; front-face geometry) was red-shifted from the 0.02 wt% CNC in N_2_-saturated n-octane (λ_ex_ = 318 nm and λ_em_ = 350 nm) by 10 and 7 nm, respectively. Furthermore, emissions from different concentrations of CNC in n-octane were found to be independent of the excitation wavelength. The nature of the nucleation and growth processes for the growing gelator assemblies were determined according to the Avrami equation (Equation (1), where X is the volume fraction of the gel, *K* is a temperature-dependent rate constant, *n* is the Avrami exponent, and *t* is time) [97,98].

(1) $$\ln\;\left(\ln\frac{1}{1-X}\right)=\ln K+n\ln t$$

For example, an Avrami plot (Figure 28b) gave a slope (n) = 1.08, suggesting aggregation involving instantaneous nucleation (zero-order) and one-dimensional growth. 

## 6. Mechanical and Other Perturbations on Emitting Gels

**Figure 29:**
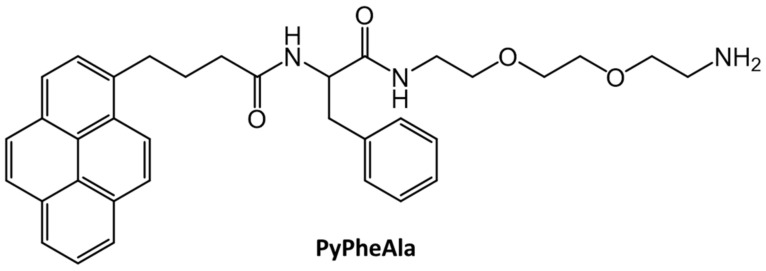
Molecular structure of PyPheAla.

Kar et al., reported the in situ synthesis of 25–40 nm silver nanoparticles by irradiating with sunlight a hydrogel containing AgNO_3_ without an extrinsic reducing agent. The gelator, PyPheAla, was comprised of pyrenyl and L-phenylalanine substituents (Figure 29) [34]. A 5.6 × 10^−4^ mM solution of PyPheAla in water upon excitation at 340 nm emitted strongly at 376, 395 and 417 nm, indicative of emission from a pyrenyl monomer. Aggregation of pyrenyl units was observed in a 2.8 mM PyPheAla hydrogel, with a new emission peak centered at 467 nm. Furthermore, the silver nanoparticles within the hydrogel enhanced its overall mechanical strength (i.e., a three-fold increase in the magnitude of the storage modulus at 0.1% strain). 

**Figure 30:**
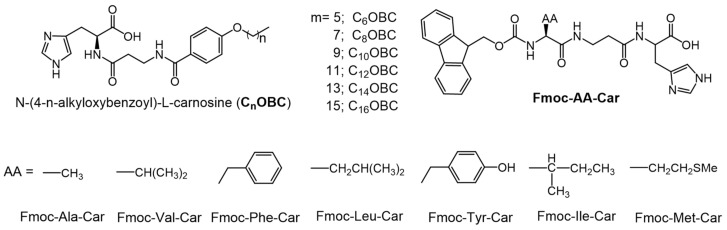
Molecular structure of C_n_OBC and Fmoc-AA-Car.

Pal et al., studied pH dependence on the hydro-gelating abilities of L-carnosine based amphiphiles with different alkoxy chain lengths [99,100]. For the series of N-(4-*n*-alkyloxybenzoyl)-L-carnosines (C_n_OBC, where n = 6–16, Figure 30), the T_g_ values, thermal stabilities and mechanical stabilities increased with alkoxy chain length due to increased van der Waals interactions. Furthermore, the CGC values of the C_n_OBC were lowest at neutral pH. At both acidic and basic pH values, the hydrogelators exist in a charged form, so that interionic repulsion makes aggregation difficult. However, the hydrogels at pH 2 exhibit higher thermal stability and lower mechanical stability than the respective gels at pH 7. In addition, Mahapatra et al., studied L-carnosine based hydrogelators which are covalently linked to Fmoc-protected amino acids, Fmoc-AA-Car, where AA is an amino acid (Figure 30) [101]. Among the gelators examined, the one with Phe as the amino acid, Fmoc-Phe-Car, had the lowest CGC (0.24% *w*:*v* at pH 2 and 0.28% *w*:*v* at pH 7) due to additional π–π stacking interactions. Fmoc-Tyr-Car exhibited the highest CGC due to strong intermolecular hydrogen bonding of phenolic -OH groups. The CGC values of Fmoc-Val-Car were between the two extrema (0.26% *w*:*v* at pH 2 and 0.70% *w*:*v* at pH 7); Fmoc-Ala-Car failed to form gel at either pH. The emission maximum of gels of Fmoc-Phe-Car (λ_ex_ = 262 nm) were red-shifted at pH 7 (20 nm) and at pH 2 (13 nm) with respect to the λ_em_ = ~315 nm found for a 10^−6^ M aqueous solution at pH 2 and pH 7. The red-shifts are consistent with lower electrostatic repulsion and, thus, more π–π stacking interactions in the solutions. As additional support for this hypothesis, it was found that the mechanical stability and T_g_ value of the Fmoc-Phe-Car gel was higher at pH 7 than at pH 2.

## 7. Perspectives and Challenges for the Future

A comprehensive understanding of the structure–property relationships within gel networks remains a challenge that will probably persist for many years into the future [102,103]. In addition, the kinetics and thermodynamics of gel formation are still not clearly understood [104,105]. Despite these "problems", interest in the field of luminescent gels, specifically those comprised of LMOGs, has increased in the past few decades because they help scientists to understand processes such as gelation, degrees of molecular aggregation, and interactions within self-assembled networks. They provide an additional tool for investigating the specific consequences of different modes or molecular packing and how they differ from the properties of unassociated (non-luminescent) LMOGs. Thus, apart from offering a variety of potential applications, luminescent gels open several new opportunities for materials scientists.

The photophysical properties of many types of LMOGs can be easily modified and tuned by synthetic methods. However, many commercial applications (such as for drug delivery, bio-imaging, chemical sensing or inclusion in optoelectronic devices), requiring relatively large amounts of luminescent LMOGs, are currently limited by production costs, the need for high purities, and environmental and radiation stability issues. In addition, seemingly benign structural modifications to a functional luminescent LMOG, such as changing the position of the emitting functional group within a molecular frame or even changing the length of a non-luminescent part of an LMOG (e.g., the length of an alkyl chain), can drastically decrease (or increase) both the efficiency of emission and the ability of the LMOG to form a gel [106,107,108]. Additionally, there are few known examples of fluorescent or phosphorescent LMOGs which have been shown to be biocompatible. These are challenges for those interested in the field, and they offer opportunities for those with the insight and imagination to overcome the limitations.

## Abbreviations

AIEE	aggregation-induced emission enhancement
ALS	aromatic linker steroid
CCCP	carbonyl cyanide m-chlorophenylhydrazone
CGC	critical gelator concentration
DF	delayed fluorescence
T_g_	gel melting temperature
H-bonding	hydrogen bonding
HEPES	2-[4-(2-hydroxyethyl)piperazin-1-yl]ethanesulfonic acid
LMOG	low molecular-mass organic gelator
τ	lifetime
Δψ_m_	membrane potential
MT	MitoTracker Red FM
NDI	napthalene diimide
PA	picric acid
φ	quantum yield
K_sv_	Stern-Volmer quenching rate constant
TADF	thermally activated delayed fluorescence
TPE	tetraphenylethene
UV	ultraviolet
WAXS	wide angle X-ray scattering
